# Do Economic Crises Always Undermine Trust in Others? The Case of Generalized, Interpersonal, and In-Group Trust

**DOI:** 10.3389/fpsyg.2018.01955

**Published:** 2018-10-15

**Authors:** Ginés Navarro-Carrillo, Inmaculada Valor-Segura, Luis M. Lozano, Miguel Moya

**Affiliations:** ^1^Mind, Brain and Behavior Research Center, Department of Social Psychology, Universidad de Granada, Granada, Spain; ^2^Mind, Brain and Behavior Research Center, Department of Methodology for Behavioral Sciences, Universidad de Granada, Granada, Spain

**Keywords:** economic crisis, great recession, trust, generalized trust, interpersonal trust, depersonalized in-group trust

## Abstract

After the global economic collapse triggered by the Great Recession, there has been an increased interest in the potential psychological implications of periods of economic decline. Recent evidence suggests that negative personal experiences linked to the economic crisis may lead to diminished generalized trust (i.e., the belief that most of the people of the society are honest and can be trusted). Adding to the growing literature on the psychological consequences of the economic crisis, we propose that the perceived personal impact of the economic crisis not only would undermine generalized trust but also may lead to increased interpersonal trust (i.e., directed to specific and close people) and depersonalized in-group trust [i.e., directed to individuals who, while strangers, belong to the same group (e.g., social class)]. Across three studies (*N* = 1379), we tested these central hypotheses and ascertained whether the perceived personal impact of the crisis would predict these types of trust (assessed using questionnaire and behavioral measures) independent of individuals’ socioeconomic status. Non-experimental data from Study 1 revealed that a higher perceived personal impact of the crisis is related to lower levels of generalized trust and higher levels of interpersonal trust. These effects were independent of participants’ socioeconomic status. Non-experimental data from Study 2 replicated the findings obtained in Study 1 and also showed a positive association between the perceived personal impact of the crisis and depersonalized in-group trust. This pattern of results emerged even after controlling for socioeconomic status, gender, age, political orientation, religiosity, and unemployment status. In Study 3, using an experimental design, we found that the salience of a possible economic downturn led to decreased generalized trust and increased interpersonal and depersonalized in-group trust – independently of socioeconomic status – compared with the control condition. These results challenge the conventional wisdom that economic crises invariably undermine trust in others. The implications of the present research as well as future research directions are discussed.

## Introduction

When considering the gravity of its consequences, the current economic crisis, ultimately resulting from the financial crisis originating in the United States at the beginning of 2008, has been continuously equated with the Great Depression in the 1930s (Grusky et al., 2011). Although its effects are global, certain countries have been more impacted than others. In the case of Europe, countries such as Portugal, Greece, Italy, and Spain have been particularly affected by this context of widespread economic enfeeblement. In Spain, after a decade marked by economic expansion, the signs of incipient economic collapse became apparent with the fall of the housing market in 2007 (Gili et al., 2013). Since the second quarter of 2008, the GDP started to fall, thus marking the beginning of a recessionary economic period (Lopez-Bernal et al., 2013). This economic crisis, triggered partly by the international financial crisis, which had its epicenter in the United States (Carballo-Cruz, 2011), caused (and continues to cause) a marked increase in social vulnerability in Spain. For example, poverty, unemployment, and inequality have dramatically increased during this time (López-Jiménez and Renes, 2011). Concretely, in 2016, the percentage of people at risk of poverty reached 22.3% (Instituto Nacional de Estadística, 2017a). In the case of unemployment, the national average in 2007, that is, before the crisis, was 8.42%, while 10 years later, it was 18.75% (Instituto Nacional de Estadística, 2017b). Moreover, Spain is one of the European countries that has experienced the largest increase in inequality since the beginning of the recession (in 2015, the richest 1% of the Spanish population owned as much wealth as the poorest 80%; Oxfam Intermon, 2016).

Apart from its economic consequences, the crisis affects many areas of social life, rendering the study of its psychosocial effects undeniably relevant. However, scarce empirical evidence on the psychological consequences of this period of economic instability is available. The relationship between the perceived personal impact of the economic crisis and trust in others, a central social motivation (Fiske, 2004), is analyzed in this article. In our research, we examine whether the perceived economic threat related to the Spanish economic crisis affects different types of trust (generalized trust, interpersonal trust, and depersonalized in-group trust). More specifically, we test the hypothesis that greater perceived personal impact of the crisis affects trust in others differentially. We postulate that greater perceived impact of the economic crisis would undermine generalized trust on the one hand and increase interpersonal trust and depersonalized in-group trust levels on the other.

### Trust

Trust can be defined in many ways, mainly depending on the discipline from which it is studied. Psychologists tend to define it as a set of interrelated cognitive processes, attitudes, and beliefs regarding a psychological state of vulnerability or risk derived from the uncertainty associated with the future intentions and actions of those on whom one depends (Kramer, 1999). Thus, some definitions of trust emphasize the expectations and predictability of the behavior of other individuals during social interactions (McAllister, 1995). In this line, Rousseau et al. (1998) defined trust as “a psychological state comprising the intention to accept vulnerability based upon positive expectations of the intentions or behavior of another” (p. 395). Other authors state that trust includes expectations regarding the good intentions of other people in situations singled out by a conflict between collective interests and the self (Yamagishi, 2011). Following this author, trust includes expectations of experiencing behaviors that are favorable to our interests in a social situation characterized by uncertainty. It is evident from the above that in contexts that favor vulnerability and uncertainty, trust (i.e., expectations regarding how others will behave toward us) plays a crucial role (Simpson, 2007).

Trusting others is essential for myriad reasons. Trust promotes the development of mutual cooperation and results in a greater commitment to and satisfaction with relationships (Balliet and Van Lange, 2013; Fitzpatrick and Lafontaine, 2017). It also improves the conditions of everyday life at the individual and interpersonal levels because people with greater trust are more successful in the social sphere and tend to be less vengeful, resentful, and solitary in comparison to those who trust others to a lesser extent (see Fiske, 2004). Trust is also associated with lower levels of social anxiety (Kaplan et al., 2015), better physical health (Schneider et al., 2011), and higher personal well-being (Helliwell and Wang, 2011). It is important to mention that the positive effects of trust in others are not limited exclusively to the individual level. For instance, in the organizational field, trust promotes cooperative behavior, reduces conflict, and favors the deployment of effective responses to crises (Rousseau et al., 1998). At the group level, it promotes cohesion among its members, whereas at the social level, a society with greater levels of trust and general reciprocity is more efficient than a distrustful society (Putnam, 2000). Thus, “a nation’s well-being, as well as its ability to compete, is conditioned by a single, pervasive cultural characteristic: the level of trust inherent in the society” (Fukuyama, 1995, p. 7). Consistent with the foregoing, Knack and Keefer (1997) found a positive correlation between trust and GDP. Similarly, Bjørnskov and Méon (2013) indicated that trust determines the quality and proper functioning of legal and bureaucratic institutions. Thus, trust relates to optimal personal and social functioning.

Much of the literature on trust has traditionally focused on the study of the determinants and consequences of a particular type of trust: generalized trust. This form of trust expresses the idea that the behavior of others, whose identity is unspecified, is honest (Fukuyama, 1995). However, it seems that when people respond to a standard survey item on this type of trust (i.e., “Generally speaking, would you say that most people can be trusted or that you need to be very careful in dealing with people?”), they are usually thinking about out-group members (Delhey et al., 2011). Interest in studying other types of trust, such as interpersonal (or particularistic) trust and depersonalized in-group trust (e.g., Kenworthy and Jones, 2009; Fiske et al., 2012; Fritsche et al., 2017; Navarro-Carrillo et al., 2018), has recently increased. The latter form of trust (i.e., depersonalized in-group trust) is directed toward individuals who, while strangers, belong to common categories. Numerous situations in different social contexts entail trusting (or not) individuals regarding whom we have little information. In this scenario, knowing that a person (or persons) belongs to our in-group could tilt the balance in favor of trusting, even if this fact is the only thing we know about them (see Kenworthy and Jones, 2009). However, while generalized trust is directed to a stranger, that is, someone regarding whom we have no information and with whom we have no relationships, interpersonal trust relates to close people with interpersonal relationships (e.g., acquaintances, family, friends, etc.; Yamagishi and Yamagishi, 1994; Navarro-Carrillo et al., 2018). By being primarily focused on someone you are close with and know well, this trust facilitates defining with greater accuracy the possible response of these types of partners toward oneself, thus decreasing social uncertainty (Yamagishi et al., 1999). Igarashi et al. (2008) established a distinction between the consequences of particularistic (i.e., interpersonal) and generalized trust. For these authors, particularistic trust can help people maintain social relations once they have been created because the emotional connection typical of this trust may help in maintaining secure and committed social relations. By contrast, generalized trust promotes the approach and nearness to other people to create social relations, regardless of whether prior interpersonal relationships exist. Therefore, because most people are considered to be worthy of trust, there should be no difficulty in establishing relations with them, expanding beyond the circles formed by relatives and friends and, in this manner, enabling economic and social opportunities (Yamagishi et al., 1998). In this regard, Van Lange (2015) suggests that generalized trust plays a central role in promoting, establishing, and maintaining cooperation. In short, although interpersonal trust may reinforce the commitment to social relations, it will most likely not encourage people to create new relations and seek new social opportunities.

### Economic Crisis and Trust

Different studies have shown that increasing levels of economic inequality, one of the striking consequences of the economic crisis (Organization for Economic Cooperation and Development [OECD], 2014), are negatively related to generalized trust (e.g., Wilkinson and Pickett, 2009; Buttrick and Oishi, 2017). However, there is little research on the direct consequences of suffering the effects of the economic crisis on generalized trust. The few studies that exist have shown that adverse personal experiences related to the financial crisis contribute to reducing generalized trust (van der Cruijsen et al., 2016). Indeed, groups confront one another when resources seem to be scarce, and as a likely result, they trust each other less (Fiske et al., 2012). In addition, given that trust involves a degree of risk due to the possible betrayal by others (Yamagishi, 2011), it makes sense to think that those people most threatened by the crisis may have lower levels of generalized trust. Additionally, a positive relationship between social class and generalized trust has been found (e.g., Alesina and La Ferrara, 2002; Gheorghiu et al., 2009), which suggests that economically favorable contexts favor generalized trust, while unfavorable ones do the opposite.

However, will the economic crisis affect interpersonal and in-group trust in much the same way that it would affect generalized trust? To the best of our knowledge, there is no research that directly analyzes these effects. Nonetheless, there is indirect evidence that permits us to infer that the effects of the economic crisis on these types of trust will not develop in the same direction as they do for generalized trust. Thus, for example, it has been reported that interpersonal trust and in-group trust (within one’s own social class) were higher among individuals with lower socioeconomic resources (Navarro-Carrillo et al., 2018). People affected by the economic crisis are able to access a smaller network of economic and material support, which translates into a greater need for social support and security. Consequently, it is likely that they experience increased levels of interpersonal trust, which, in turn, provides certainty and stability (Yamagishi and Yamagishi, 1994). That is, people who are more adversely affected by the crisis may tend to maintain safe interpersonal relations with close and familiar people to reduce uncertainty and ensure the protection of their limited resources in a general context of vulnerability and risk. In a similar vein, trusting people who belong to our in-group may help mitigate the negative consequences arising from states of personal uncertainty (Hogg, 2007). Therefore, this type of trust may increase when an individual is negatively affected by the economic crisis (Fritsche et al., 2017).

In short, we hypothesize that greater perceived personal impact of the crisis would relate with lower generalized trust on the one hand and with greater interpersonal and in-group class trust on the other. These hypotheses are tested in three studies: one with a university sample (Study 1) and two with general population samples (Studies 2 and 3). In the first two, we used a non-experimental design and assessed trust through questionnaires, whereas in the third, we followed an experimental strategy and examined trust through behavioral measures (i.e., a trust game).

## Study 1

This study aimed to verify the expected pattern of relationships between the perceived personal impact of the economic crisis and trust in others (generalized, interpersonal, and depersonalized in-group). To rule out possible alternative explanations for the results and given that previous studies have shown socioeconomic status to be associated with these trust types (Navarro-Carrillo et al., 2018), we also sought to determine if the hypothesized relationships existed independently of the participants’ socioeconomic status.

### Materials and Methods

#### Participants

Three hundred sixty-seven individuals (270 female, 91 men, 6 unreported) with a mean age of 21.26 years (*SD* = 3.78; range from 18 to 50) who were pursuing university studies at the time participated in this study. None of the participants quit the study (**Table 1** displays socio-demographic characteristics for participants’ socioeconomic status).

**Table 1:** Demographic information corresponding to participants’ socioeconomic status across all studies.

Variable	Study 1		Study 2		Study 3	
	*n*	%	*n*	%	*n*	%
Family income						
<1.000€	45	12.3	279	34.4	23	11.5
1.000€–2.000€	139	37.9	328	40.4	85	42.5
2.000€–3.000€	82	22.3	123	15.1	46	23
3.000€–4.000€	49	13.4	47	5.8	19	9.5
4.000€–5.000€	15	4.1	16	2	11	5.5
>5.000€	13	3.5	19	2.3	4	2
Not reported	24	6.5	–	–	12	6
Maternal education						
Primary school	75	20.4				
Secondary education/school graduate	87	23.7				
Vocational training	55	15				
High school/diploma	29	7.9				
University not completed	14	3.8				
University completed	98	26.7				
Doctorate	–	–				
Not reported	9	2.5				
Paternal education						
Primary school	82	22.3				
Secondary education/school graduate	62	16.9				
Vocational training	48	13.1				
High school/diploma	44	12				
University not completed	24	6.5				
University completed	97	26.4				
Doctorate						
Not reported	10	2.7				
Participant education						
Primary school			35	4.3	19	9.5
Secondary education/school graduate			87	10.7	25	12.5
Vocational training			122	15	22	11
High school/diploma			59	7.3	26	13
University not completed			129	15.9	34	17
University completed			367	45.2	71	35.5
Doctorate			13	1.6	–	–
Not reported			–	–	3	1.5

#### Procedure

Two trained evaluators requested the participants’ collaboration and informed them of the estimated duration (approximately 15 min) and the strict compliance with the principles of confidentiality and anonymity regarding their responses. The sample was obtained through an incidental sampling procedure. The participants completed the questionnaire booklet individually and voluntarily in different study centers and study areas belonging to a university located in a southeast Spanish city under the direct supervision of the evaluators. The city that is home to this university, with approximately 236,000 inhabitants, is located in one of the Spanish regions most affected by the economic crisis [its unemployment rate in 2017 was 25.66% (Instituto Nacional de Estadística, 2017c), which is significantly higher than that in 2007 (10.60%), i.e., prior to the crisis]. All of the participants were undergraduate students and were volunteers, so no monetary or academic incentives were provided for participation. Finally, participants were fully debriefed and thanked. This study is part of a research project approved by the Ethical Committee of the University of Granada. All participants provided informed written consent in accordance with the Declaration of Helsinki.

#### Measures

##### Generalized trust

We used the Yamagishi and Yamagishi (1994) General Trust Scale. This instrument consists of a total of six items (e.g., “Most people are basically honest”), with a five-point Likert-type response format ranging from 1 (*strongly disagree*) to 5 (*strongly agree*). All the items of this scale are worded in a positive fashion. An average score was calculated. Higher scores in this measure reflect higher levels of generalized trust (α = 0.71).

##### Interpersonal trust

A measure that assesses trust in close people was administered (Moya et al., 2011). It consists of five items (e.g., “I only trust the people who I know personally”). Scores were provided on a five-point Likert scale ranging from 1 (*strongly disagree*) to 5 (*strongly agree*). All the items of this instrument are worded in a positive fashion. An average score was calculated. Higher scores indicate a greater interpersonal trust (α = 0.74).

##### Depersonalized in-group trust

We used a 3-item measure developed by Kenworthy and Jones (2009), adapted to participants’ social class as the in-group (e.g., “I trust all members of my social class background”), in addition to a further item (“For me, everyone who belongs to my social class is trustworthy”). The answer format is a Likert-type scale with five options ranging from 1 (*totally disagree*) to 5 (*totally agree*). As in the case of the previous instruments, this measure does not include reversed items. An average score was calculated. Higher scores reflect increased depersonalized in-group trust (α = 0.80).

##### Socioeconomic status (SES)

Participants’ SES was operationalized based on family income and parental education (Kraus et al., 2009). Monthly family income was coded into six categories: (a) under 1.000€; (b) 1.000–2.000€; (c) 2.000–3.000€; (d) 3.000–4.000€; (e) between 4.000 and 5.000€; and (f) over 5.000€. We assigned a code number from 1 to 6 to each category. Thus, higher numbers indicated greater family income. Parental educational attainment was coded into seven categories: (a) Primary school; (b) secondary education/school graduate; (c) vocational training; (d) high school/diploma; (e) university not completed; (f) university completed; and (g) doctorate. Educational level was scored from 1 to 7, with higher numbers indicating greater parental educational attainment. Lastly, scores on family income and parental educational level were standardized and summed to obtain a composite measure of SES.

##### Perceived impact of the economic crisis

A measure that assesses whether people descended in the social hierarchy due to the economic crisis was administered (Fritsche et al., 2017): “Faced with the current economic situation and thinking about your and your family’s situation, do you believe that the economic crisis has made you descend in the social scale? Please mark the option that best reflects your current situation”. The items used were as follows: (1) *Yes*, *I used to be in the upper class*, *and now I am in the upper-middle class*; (2) *Yes*, *I used to be in the upper-middle class*, *and now I am in the middle class*; (3) *Yes*, *I used to be in the middle class*, *and now I am in the lower-middle class*; (4) *Yes*, *I used to be in the lower-middle class*, *and now I am in the lower class*; (5) *Yes*, *I used to be in the lower class*, *and now I am in a very delicate situation*, *dreading a fall into poverty*; (6) *No*, *the crisis has not made me descend in the social scale;* (7) *No, the crisis has made me ascend in the social scale*; and (8) *I prefer not to answer*. Two groups were created from the responses obtained: the first comprised those who reported having descended in the social ladder (response categories 1–5) and the second comprised those who had not undergone any changes or had climbed the social scale (response categories 6–7). **Table 2** lists the frequencies corresponding to descending/not descending in the social class hierarchy during the crisis.

**Table 2:** Frequencies of descending/not descending in the social scale as a result of the economic crisis.

Faced with the current economic situation and thinking about your and your family’s situation, do you believe that the economic crisis have made you descend in the social scale?	*n*	%
1. Yes, I used to be in the upper class, and now I am in the upper-middle class.	2	0.5
2. Yes, I used to be in the upper-middle class, and now I am in the middle class.	61	16.6
3. Yes, I used to be in the middle class, and now I am in the lower-middle class.	82	22.3
4. Yes, I used to be in the lower-middle class, and now I am in the lower class.	18	4.9
5. Yes, I used to be in the lower class, and now I am in a very delicate situation, dreading a fall into poverty.	5	1.4
6. No, the crisis has not made me descend in the social scale.	184	50.1
7. No, the crisis has made me ascend in the social scale.	2	0.5
8. I prefer not to answer.	6	1.6
Not reported.	7	1.9
Personal socioeconomic descent (1–5)	168	45.7
Non-descent (6–7)	186	50.6

### Results

#### Descending/Not Descending in the Social Ladder as a Result of the Economic Crisis

As shown in **Table 2**, nearly half of the participants (45.7%) descended the social ladder due to the economic crisis. Conversely, 50.6% of participants expressed not having descended the social ladder as a consequence of the crisis.

#### Descriptive Statistics and Bivariate Correlations

**Table 3** presents the descriptive statistics and the bivariate correlations between measures of trust, SES, and perceived personal impact of the crisis. As shown, SES was significantly (and negatively) associated with depersonalized in-group trust. On the other hand, the perceived impact measure significantly correlated with generalized trust and interpersonal trust, although it was not significantly related to depersonalized in-group trust. Specifically, participants who perceive themselves as adversely affected by the crisis reported lower levels of generalized trust and higher levels of interpersonal trust. Generalized trust was negatively associated with interpersonal trust and positively with depersonalized in-group trust; interpersonal trust and depersonalized in-group trust were positively correlated.

**Table 3:** Descriptive statistics and bivariate correlations between measures of generalized trust, interpersonal trust, depersonalized in-group trust, socioeconomic status, and perceived impact of the crisis (Study 1).

Variable	*M*	*SD*	1	2	3	4	5
(1) Generalized trust	2.98	0.55	-				
(2) Interpersonal trust	3.18	0.72	-0.36^***^	-			
(3) In-group SES trust	1.65	0.67	0.21^***^	0.12^*^	-		
(4) SES	-	-	0.06	-0.01	-0.32^***^	-	
(5) Perceived impact EC^a^	-	-	0.11^*^	-0.12^*^	0.04	0.17^**^	-


*SES, socioeconomic status; EC, economic crisis. ^a^Economic descent = 0, non-economic descent = 1. ^∗^p < 0.05; ^∗∗^p < 0.01; ^∗∗∗^p < 0.001.*

#### Effect of the Perceived Impact of the Economic Crisis on the Different Types of Trust

MANCOVA was performed, involving an independent variable with two levels (perceived impact of the economic crisis: descending and maintaining/ascending the social ladder), generalized trust, interpersonal trust, and depersonalized in-group trust as dependent variables, and the composite measure of participants’ SES as a covariate.

The multivariate test results were significant for the perceived impact of the crisis, *F*(3,320) = 3.26, *p* = 0.022, Wilks’ λ = 0.970, η^2^ = 0.030, and for SES, *F*(3,320) = 16.63, *p* < 0.001, Wilks’ λ = 0.865, η^2^ = 0.135. More specifically, the results revealed a main effect of the perceived impact of the economic crisis on generalized trust, *F*(1,322) = 4.26, *p* = 0.040, η^2^ = 0.013. Participants who descended the social ladder as a result of the crisis had lower levels of generalized trust (*M* = 2.90, *SD* = 0.59) compared with participants who were not adversely affected by the crisis (*M* = 3.04, *SD* = 0.52). The analysis also showed a main effect of the perceived impact of the economic crisis on interpersonal trust, *F*(1,322) = 4.92, *p* = 0.027, η^2^ = 0.015, indicating greater interpersonal trust among those participants who fell in the social hierarchy due to the crisis (*M* = 3.29, *SD* = 0.71) compared to those who maintained or climbed positions (*M* = 3.11, *SD* = 0.73). No differences were found in depersonalized in-group trust, *F*(1,322) = 3.29, *p* = 0.071, η^2^ = 0.010, based on the perceived impact of the economic crisis.^1^ Concerning the effects of the covariate (i.e., SES), the results did not show a significant effect of SES on generalized trust, *F*(1,322) = 0.49, *p* = 0.483, η^2^ = 0.002, or on interpersonal trust, *F*(1,322) = 0.07, *p* = 0.789, η^2^ < 0.001. They showed a significant effect of the covariate on depersonalized in-group trust, *F*(1,322) = 39.34, *p* < 0.001, η^2^ = 0.109.

### Discussion

Although it has been suggested that periods of crisis or economic instability undermine generalized trust (e.g., van der Cruijsen et al., 2016), little is known regarding how economic crises or economic downturns may affect other types of personal trust. The results of this study provide promising support for the hypothesis that the perceived personal impact of the economic crisis is differentially linked with trust in others depending on the “trustee” considered. Specifically, the more that participants are affected by the crisis, the lower their generalized trust and the greater their trust in people they know (i.e., interpersonal trust). In addition, the data showed that this effect is independent from the participants’ SES. Although other studies have reported differences in in-group social class trust depending on whether the crisis affected them with greater or lesser intensity (Fritsche et al., 2017), no such differences were found in this case. One possible explanation is that belonging to this specific group (i.e., social class), in spite of constituting a relevant category in times of economic hardship (Moya and Fiske, 2017), may be barely relevant for the university population. Also, it is worth mentioning that, even though the perceived impact measure used captures whether participants have descended (or not) in the social hierarchy, it does not allow assessing whether they have fallen more than one level due to the crisis. This last fact, as well as the status of the participants, leads us to consider the need for similar research on the general Spanish population.

## Study 2

This study aimed to replicate the results of Study 1 in a large and diverse sample of the Spanish general population. In addition to controlling for SES, we controlled for other socio-demographic and ideological factors likely to affect trust, such as marital status, religion, and political orientation. In this regard, previous research has found that individuals’ political ideology can modulate their trust (Hernandez and Minor, 2015) and that propensity to trust others is greater among married men and women (Lindström, 2012) and non-religious people (Berggren and Bjørnskov, 2011). In addition, given that the increase in unemployment is one of the most important consequences of the economic crisis in Spain (López-Jiménez and Renes, 2011), we also included this variable in the regression equations. By accounting for several socio-demographic and ideological characteristics, we aimed at determining the unique contribution of the perceived personal impact of the economic crisis to trust in others.

### Materials and Methods

#### Participants

A total of 1030 participants were recruited. Of those participants, 218 were not included in the analyses because, although they accessed the survey, they did not complete all of the main measures and quit the survey during the task. Thus, our final sample included 812 Spanish adults (584 women and 228 men) aged between 18 and 64 years (*M* = 31.74, *SD* = 9.53). Most participants were single (68%); 25.9% were married; 5.7% were divorced; and 0.5% widowers. Regarding occupation, the majority of the sample consisted of employed people (42.4%), followed by unemployed (32.3%), students (24%), and pensioners (1.4%). The demographic characteristics for the SES of the participants can be found in **Table 1**.

#### Procedure

The participants were provided with the same information as those in Study 1. In this case, however, the participants completed an online questionnaire. This questionnaire was disseminated through different networks and social platforms (e.g., Facebook). In this study, the participants entered a 100€ prize drawing by completing the questionnaire online. Finally, after thanking them for their participation, we provided the participants with the e-mail address of one of the researchers responsible for the study in case they required additional information. The present study is also part of a research project approved by the Ethical Committee of the University of Granada. All participants gave informed written consent in accordance with the Declaration of Helsinki.

#### Measures

##### Generalized trust, interpersonal trust, and depersonalized in-group trust

The measures of generalized trust (α = 0.73), interpersonal trust (α = 0.66), and depersonalized in-group trust (α = 0.73) were identical to those administered in the previous study.

##### Political ideology

The participants indicated their political orientation in a Likert-type scale ranging from 0 (*left wing*) to 10 (*right wing*).

##### Religiosity

Participants reported the degree to which they considered themselves to be religious using a Likert-type scale whose values ranged from 1 (*not religious*) to 7 (*extremely religious*).

##### Socioeconomic status (SES)

Socioeconomic status was examined in the same way as in Study 1. However, in this study, this variable was operationalized taking into account the participants’ educational level in addition to monthly family income. These two scores were standardized. Then, a sum score was calculated.

##### Perceived impact of the economic crisis

The Financial Threat Scale (FTS; Marjanovic et al., 2013) was used. This instrument, adapted to the current context of the economic crisis in Spain (“Considering the economic crisis in Spain, indicate how you feel regarding your current financial situation by answering the following questions”), evaluates the participants’ perceived threat in relation to their current personal finances. It comprises a total of five items (e.g., “How much do you feel at risk?”) in a Likert-type response format, with scores ranging from 1 (*not at all*) to 5 (*a great deal*). This scale was used instead of the one used in Study 1 as a design improvement. The FTS has the proper number of alternatives (Lozano et al., 2008), and all the items are worded in a positive fashion, which produces better psychometric properties in the scale (Suárez-Álvarez et al., 2018). An average score was calculated. Higher scores mean a greater perceived personal impact of the economic crisis (α = 0.88).

### Results

#### Descriptive Statistics and Bivariate Correlations

**Table 4** presents the descriptive statistics and correlation patterns among the different types of trust, political ideology, religiosity, SES, and perceived impact of the economic crisis. SES was positively correlated with generalized trust. The lower that the SES of a participant was, the greater the perceived personal impact of the economic crisis. In addition, as participants felt more affected by the crisis, they showed lower generalized trust and higher interpersonal trust and depersonalized in-group trust. As in Study 1, generalized trust and depersonalized in-group trust correlated positively. We also found a positive correlation between interpersonal trust and depersonalized in-group trust. Finally, although the association between generalized trust and interpersonal trust was negative, it did not reach statistical significance.

**Table 4:** Descriptive statistics and bivariate correlations between measures of generalized trust, interpersonal trust, depersonalized in-group trust, political orientation, religiosity, socioeconomic status, and perceived impact of the crisis (Study 2).

Variable	*M*	*SD*	1	2	3	4	5	6	7
(1) Generalized trust	2.84	0.66	-						
(2) Interpersonal trust	2.99	0.74	-0.06	-					
(3) In-group SES trust	1.82	0.75	0.25^***^	0.22^***^	-				
(4) Political orientation	4.19	2.11	-0.02	0.04	-0.08^*^	-			
(5) Religiosity	3.39	2.46	0.05	0.03	-0.02	0.42^***^	-		
(6) SES	-	-	0.15^***^	-0.02	-0.06	0.06	-0.03	-	
(7) Perceived impact EC	3.45	0.98	-0.16^***^	0.09^*^	0.10^**^	-0.10^**^	-0.07	-0.26^***^	-

*SES, socioeconomic status; EC = economic crisis. ^∗^p < 0.05; ^∗∗^p < 0.01; ^∗∗∗^p < 0.001.*

#### Effect of the Perceived Impact of the Economic Crisis on the Different Types of Trust

To examine the perceived impact of the economic crisis on the different types of trust (generalized, interpersonal, and depersonalized in-group) and whether this effect was independent from certain socio-demographic and ideological characteristics, three hierarchical multiple regression analyses (one for each type of trust as criterion variables) were performed. In the first step of the analyses, the following variables were included: gender (0 = woman, 1 = man), age, marital status (0 = single, 1 = married), political ideology, religiosity, SES, and, finally, employment status (0 = not unemployed, 1 = unemployed). In the second step, the perceived personal impact of the economic crisis was introduced.

##### Generalized trust

Model 1, which included the different socio-demographic and ideological variables, was significant, *F*(7,795) = 5.501, *p* < 0.001. Specifically, the higher the age (β = 0.114, *p* = 0.005) and SES (β = 0.164, *p* < 0.001), the greater the generalized trust was (**Table 5**). Model 2, *F*(8,794) = 6.427, *p* < 0.001, showed that a higher perceived personal impact of the economic crisis was indicative of lower generalized trust (β = -0.131, *p* < 0.001). The inclusion of this variable in the regression equation explained an additional 1.5% of the variance in levels of generalized trust, *F*(1,794) = 12.358, *p* < 0.001.

**Table 5:** Summary of hierarchical regression analysis for variables predicting generalized trust (Study 2).

Predictor	Model 1			Model 2		
	β	CI (95%)	*p*	β	CI (95%)	*p*
Step 1						
Gender^a^	-0.042	[-0.162, 0.039]	0.230	-0.053	[-0.179, 0.022]	0.126
Age	0.114	[0.023, 0.128]	0.005	0.113	[0.022, 0.127]	0.005
Marital status^b^	0.030	[-0.077, 0.166]	0.471	0.014	[-0.100, 0.142]	0.735
Political orientation	-0.066	[-0.094, 0.006]	0.086	-0.072	[-0.098, 0.002]	0.059
Religiosity	0.067	[-0.006, 0.095]	0.083	0.064	[-0.008, 0.092]	0.100
SES	0.164	[0.037, 0.096]	<0.001	0.140	[0.027, 0.087]	<0.001
Employment status^c^	-0.013	[-0.122, 0.084]	0.720	0.020	[-0.078, 0.134]	0.607
Step 2						
Perceived impact EC				-0.131	[-0.135, -0.038]	<0.001
*R*^2^	0.046			0.061		
Adjusted *R*^2^	0.038			0.051		
*F*	5.501^***^			6.427^***^		
Δ*R*^2^				0.015		
Δ*F*				12.358^***^		

*SES, socioeconomic status; EC, economic crisis. ^a^Woman = 0, Man = 1; ^b^Unmarried = 0, Married = 1. ^c^Not unemployed = 0, Unemployed = 1. ^∗^p < 0.05; ^∗∗^p < 0.01; ^∗∗∗^p < 0.001.*

##### Interpersonal trust

As with generalized trust, socio-demographic and ideological variables significantly predicted interpersonal trust, *F*(7,795) = 2.686, *p* = 0.009 (**Table 6**). More specifically, younger (β = -0.134, *p* = 0.001) and married people (β = 0.099, *p* = 0.018) had greater interpersonal trust. In the second step, the incorporation of the perceived personal impact of the economic crisis in the regression equation explained an additional 0.9% of the variance in interpersonal trust, *F*(1,794) = 7.440, *p* = 0.007. The model significantly predicted interpersonal trust, *F*(8,794) = 3.300, *p* = 0.001 (**Table 6**, Model 2). Greater perceived impact of the crisis was associated with greater interpersonal trust (β = 0.103, *p* = 0.007).

**Table 6:** Summary of hierarchical regression analysis for variables predicting interpersonal trust (Study 2).

Predictor	Model 1			Model 2		
	β	CI (95%)	*p*	β	CI (95%)	*p*
Step 1						
Gender^a^	0.067	[-0.004, 0.226]	0.058	0.076	[0.011, 0.241]	0.032
Age	-0.134	[-0.159, -0.040]	0.001	-0.133	[-0.158, -0.039]	0.001
Marital status^b^	0.099	[0.029, 0.306]	0.018	0.111	[0.050, 0.327]	0.008
Political orientation	0.044	[-0.024, 0.090]	0.256	0.049	[-0.020, 0.093]	0.208
Religiosity	0.007	[-0.052, 0.063]	0.850	0.010	[-0.050, 0.065]	0.790
SES	-0.015	[-0.041, 0.027]	0.693	0.004	[-0.032, 0.036]	0.918
Employment status^c^	0.058	[-0.025, 0.211]	0.122	0.032	[-0.069, 0.173]	0.403
Step 2						
Perceived impact EC				0.103	[0.022, 0.132]	0.007
*R*^2^	0.023			0.032		
*Adjusted R*^2^	0.015			0.022		
*F*	2.686^**^			3.300^**^		
Δ*R*^2^				0.009		
Δ*F*				7.440^**^		

*SES, socioeconomic status; EC, economic crisis. ^a^Woman = 0, Man = 1; ^b^Unmarried = 0, Married = 1. ^c^Not unemployed = 0, Unemployed = 1. ^∗^p < 0.05; ^∗∗^p < 0.01; ^∗∗∗^p < 0.001.*

##### Depersonalized in-group trust

Socio-demographic and ideological characteristics did not predict depersonalized in-group trust in Model 1, *F*(7,795) = 1.837, *p* = 0.077 (**Table 7**, Model 1). However, Model 2 did so, *F*(8,794) = 2.203, *p* = 0.025. The higher that the perceived personal impact of the economic crisis was, the greater the in-group trust (β = 0.083, *p* = 0.030). In this second step, the amount of explained variance of depersonalized in-group trust increased by 0.6%. This increase was statistically significant, *F*(1,794) = 4.703, *p* = 0.030.^2^

**Table 7:** Summary of hierarchical regression analysis for variables predicting depersonalized in-group trust (Study 2).

Predictor	Model 1			Model 2		
	β	CI (95%)	*p*	β	CI (95%)	*p*
Step 1						
Gender^a^	0.071	[0.003, 0.235]	0.045	0.079	[0.014, 0.247]	0.028
Age	0.014	[-0.050, 0.071]	0.725	0.015	[-0.049, 0.072]	0.714
Marital status^b^	-0.010	[-0.157, 0.123]	0.809	<0.001	[-0.141, 0.140]	0.996
Political orientation	-0.073	[-0.113, 0.003]	0.062	-0.069	[-0.110, 0.005]	0.076
Religiosity	0.008	[-0.052, 0.064]	0.839	0.010	[-0.050, 0.066]	0.791
SES	-0.046	[-0.055, 0.013]	0.221	-0.031	[-0.049, 0.020]	0.416
Employment status^c^	0.048	[-0.043, 0.196]	0.208	0.027	[-0.079, 0.166]	0.490
Step 2						
Perceived impact EC				0.083	[0.006, 0.118]	0.030
*R*^2^	0.016			0.022		
Adjusted *R*^2^	0.007			0.012		
*F*	1.837			2.203^*^		
Δ*R*^2^				0.006		
Δ*F*				4.703^*^		

*SES, socioeconomic status; EC, economic crisis. ^a^Woman = 0, Man = 1; ^b^Unmarried = 0, Married = 1. ^c^Not unemployed = 0, Unemployed = 1. ^∗^p < 0.05; ^∗∗^p < 0.01; ^∗∗∗^p < 0.001.*

### Discussion

Study 2 fully replicates the findings obtained in Study 1 for a wide and diverse sample of the general Spanish population: the more that people feel affected by the economic crisis (measured differently from in Study 1), the lower their generalized trust and the greater their interpersonal trust. Unlike Study 1, in this case, we did find a positive relationship between the perceived impact of the crisis and trust in people of the same social class (i.e., in-group trust). This latter outcome is consistent with the findings of other research conducted in Spain (Fritsche et al., 2017). In addition, it must be stressed that relationships between being affected by the crisis and the different forms of trust emerged even when sex, age, and the other socio-demographic and ideological variables analyzed were controlled for in the regression equations. This result emphasizes the specific contribution of the perception of the personal impact of the crisis to the different types of trust. It should also be noted that Study 2 replicates the effects found in the previous study using a multi-item scale of perceived personal impact of the crisis.

Although our data consistently support our initial predictions, the non-experimental nature of Studies 1 and 2 makes it impossible to establish causality relations between the perceived impact of the economic crisis and trust. In fact, it could be that individuals’ inclination to trust in others affects how they perceive their socioeconomic context and reality. To rule out this alternative explanation, we conducted an experimental study to test the causal effects of the perceived personal impact of the economic crisis on trust.

## Study 3

Could trust in others vary momentarily if certain beliefs of the evolution of the socioeconomic environment are activated? In this study, we experimentally manipulated the salience of the economic crisis. Parallel to the previous studies, we hypothesized that activating the idea of economic downturn in the context of the crisis would result in less generalized trust and greater interpersonal and in-group trust. To make the results of Studies 1 and 2 more consistent, we measured the different types of trust in a different way; specifically, by adapting the trust game paradigm, also referred to as the investment game (Berg et al., 1995). The trust game is one of the most widely used behavioral trust measures in the literature on this subject (Johnson and Mislin, 2011). Moreover, previous research has shown that these experimental measures of trust are connected with several macro-features of nations. For instance, it has been found that higher rates of inequality, poverty, and unemployment, on the one hand, as well as lower rates of growth, on the other, are linked to lower trust exhibited in these experimental measures (see Cardenas and Carpenter, 2008). To sum up, this new design allows for expanding the approaches used in the previous studies, thus facilitating a more systematic analysis of the effects of the economic threat represented by the crisis scenario.

### Materials and Methods

#### Participants

A total of 200 Spanish adults (111 women and 89 men) between the ages of 18 and 78 years (*M* = 37.68, *SD* = 14.33) participated in the study (see **Table 1** for demographic information corresponding to participants’ SES). A total of 103 participants were randomly assigned to the declining economic condition in the context of crisis (51.5%), and 97 were assigned to the control group (48.5%). None of the participants of the two conditions who began the experiment quit the task during the course of it.

#### Procedure

Two suitably trained researchers informed the participants that this investigation aimed to analyze attitudes and opinions on different social issues. The ultimate objective of the experiment was not explicitly mentioned. The sample was obtained through an incidental sampling procedure in the same city in which the sample of the first study was obtained. The participants individually completed the questionnaire under the direct supervision of the two researchers in different public locations (e.g., local transportation stations) in the city. After completing the questionnaire, the participants were thanked and, lastly, more details on the investigation were provided. No reward was offered for participation. Like the preceding studies, this experiment is part of a research project approved by the Ethical Committee of the University of Granada. All participants provided informed written consent in accordance with the Declaration of Helsinki.

#### Measures

##### Manipulation

To manipulate the perception of the economic crisis in Spain (economic decline vs. control), we used two pieces of news from the digital edition of a Spanish newspaper specialized in economic information. Under the condition of economic decline, the fictional news emphasized that, in accordance with what was established by specialized international economic reports, the crisis situation would worsen in Spain (e.g., “*… In addition, the economy will continue destroying jobs at an alarming rate so that the crisis will continue to have a devastating effect on the real economy, reducing significantly the spending and consumption capacity of Spanish people*”). In contrast, in the control condition, the participants read news regarding “*the use of a novel analysis technique on data from the Kepler telescope*”. Similar procedures have been used by other researchers (e.g., Blanton et al., 2001; Mauss et al., 2011). To strengthen the manipulation, the participants were also asked to briefly describe the situation of someone close who could be affected by what was described in the news.

##### Manipulation check

To ensure that the experimental manipulation functioned adequately, the participants were asked about the possible evolution of the Spanish economic situation in response to what was stated in the previously presented news (i.e., “The economic situation is going to...”). A Likert-type response format with alternative responses ranging between 1 *(get worse)* and 7 (*improve*) was used.

##### Trust

To assess generalized, interpersonal, and depersonalized in-group trust, an adaptation of the trust game paradigm was used (Berg et al., 1995). During this task, which was performed three times (once for each type of trust), the participants played the role of “trustor” (the presentation of the different trust games was counterbalanced to avoid possible effects of order). The participants were asked to imagine that they participate in a game in which they initially receive 100€. Then, they were provided with information regarding the so-called “trustee”. In the generalized trust scenario, the participants were informed that the trustee was “*someone completely unknown to you*”. For the interpersonal trust scenario, the participants were told that the trustee was “*someone with whom you have a personal relationship and that you know personally*”. In the depersonalized in-group trust scenario, the participants were informed that the trustee was “*someone who belongs to your social class”.* Based on recent literature indicating that different measures of trust are linked to behavior exhibited in the trust game and several social dilemmas (e.g., Evans and Revelle, 2008; Balliet and Van Lange, 2013), we consider that the described trust scenarios are parallel behavioral measures of the trust scales administered in the previous studies. In each situation, the participants had to choose between two options regarding how to manage the 100€: either to accept 30€ out of 100€ for certain or to send the entire sum of money received (100€) to the other person (i.e., trustee). In the latter case, the trustee would decide how much he or she would keep and how much he or she would return to the trustor. Thus, the choice to send the 100€ to the other person represents the cooperation tendency/participants’ levels of trust (generalized, interpersonal, or depersonalized in-group). In sum, in this experiment, the trust game was performed as a form of survey (Xin et al., 2016).

##### Socioeconomic status (SES)

Socioeconomic status was evaluated as in Study 2, that is, considering the monthly family income and the educational background of the participants.

### Results

#### Manipulation Check

The ANOVA results, in which experimental manipulation was included as a between-group factor and the expected evolution of the Spanish economic situation as the dependent variable, revealed that manipulation functioned properly, *F*(1,194) = 33.50, *p* < 0.001, η^2^ = 0.147. The participants in the crisis-salience group believed to a greater extent that the economic situation was going to become worse (*M* = 2.53, *SD* = 1.61) compared with the control group (*M* = 3.78, *SD* = 1.42), whereby lower scores indicated “*economic deterioration*” and higher scores “*economic improvement*”.

#### Effect of the Salience of the Economic Crisis on Trust

##### Generalized trust

**FIGURE 1:**
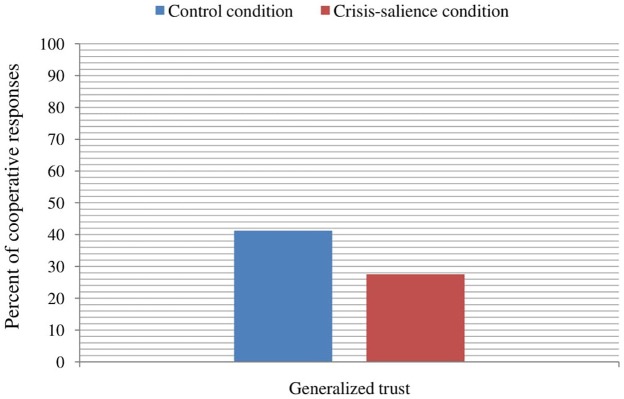
Percent of cooperative responses toward an unknown person (generalized trust) depending on the experimental condition.

To test the prediction that the salience of the crisis-related economic threat results in lower generalized trust (and to determine whether this effect is independent from SES), a binary logistic regression with two predictors [experimental manipulation (0 = control, 1 = economic deterioration) and SES] was performed using the measure of generalized trust as a criterion. Our results showed a significant main effect of experimental manipulation, *B* = -0.84, Wald = 6.89, *p* = 0.009, odds ratio (OR) = 0.43. This outcome indicates that the participants in the declining economic condition were less likely to cooperate (i.e., to send the 100€) with strangers than the participants in the control condition (**Figure 1**). SES was not significant in the model, *B* = 0.16, Wald = 2.78, *p* = 0.096, OR = 1.18. The order of the trust games’ presentation did not affect participants’ responses to the generalized trust scenario, χ^2^(2,199) = 4.20, *p* = 0.123.

##### Interpersonal trust

**FIGURE 2:**
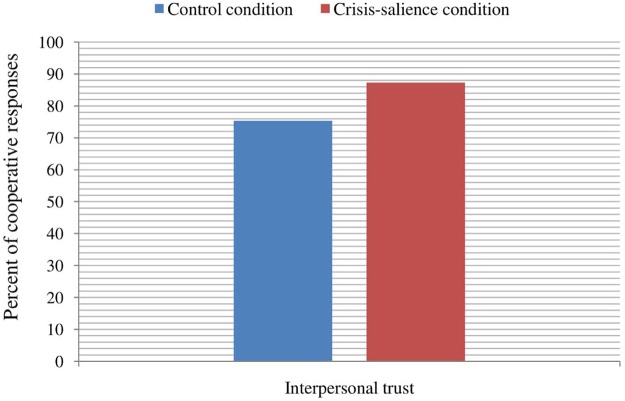
Percent of cooperative responses toward a close person (i.e., interpersonal trust) depending on the experimental condition.

As in the case of generalized trust, we conducted a similar binary logistic regression but with the tendency to cooperate with acquaintances (i.e., interpersonal trust) as a criterion variable. The results revealed a significant main effect of experimental manipulation, *B* = 0.90, Wald = 5.00, *p* = 0.025, OR = 2.47, indicating that the participants in the declining economic condition tended to cooperate (i.e., to send the 100€) more with acquaintances than the participants in the control condition (**Figure 2**). No significant effect of SES emerged in the model, *B* = 0.08, Wald = 0.43, *p* = 0.513, OR = 1.08. We did not find an effect of the order of the trust games’ presentation in the interpersonal trust scenario, χ^2^(2,199) = 5.89, *p* = 0.053.

##### Depersonalized in-group trust

**FIGURE 3:**
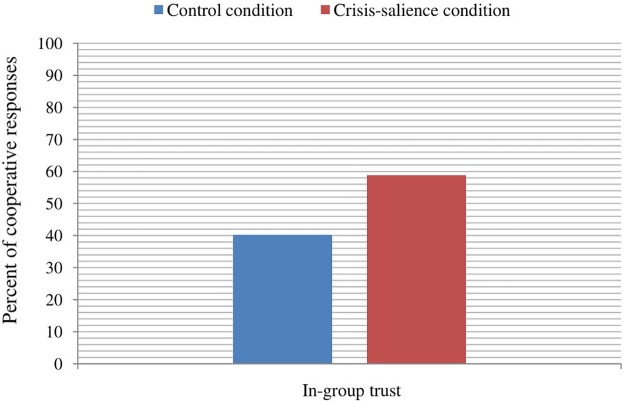
Percent of cooperative responses toward an unknown person who belongs to the same social class (i.e., depersonalized in-group trust) depending on the experimental condition

Finally, regarding trust in strangers with the same SES (i.e., depersonalized in-group trust) as a criterion variable, the results showed a significant main effect of experimental manipulation, *B* = 0.59, Wald = 3.85, *p* = 0.050, OR = 1.80, reflecting that participants in the declining economic condition were more inclined to cooperate (i.e., to send the 100€) with strangers with whom they shared SES than participants in the control condition (**Figure 3**). As in the previous cases, the effect of SES was not significant, *B* = 0.15, Wald = 2.71, *p* = 0.100, OR = 1.16. In addition, the order of the trust games’ presentation did not affect participants’ responses to the in-group trust scenario, χ^2^(2,199) = 3.99, *p* = 0.136.

### Discussion

Study 3 provides innovative experimental evidence for the idea that greater perceived personal impact of the crisis is associated, on the one hand, with lower generalized trust and, on the other hand, with greater interpersonal trust and depersonalized in-group trust. Concretely, the results of this study show that mere activation of the salience of the economic crisis through a text that indicates the possible decline of the economic crisis situation leads to a decreased tendency to cooperate with strangers (i.e., generalized trust) and an increasing tendency to cooperate with close people (i.e., interpersonal trust) and unknown people who belong to a same class (i.e., depersonalized in-group trust) compared with the control group. Therefore, this study provides empirical evidence for a causal relationship between the perceived impact of the economic crisis and trust in others.

## General Discussion

The main goal of our research is to increase knowledge regarding the interrelationship between a macrosocial factor, e.g., the economic crisis in Spain, and psychological variables, represented in this case by different forms of trust. Generally, our findings are part of an increasing literature that shows that periods of economic crisis and instability have important psychological consequences for affected populations. Our findings provide empirical evidence that challenges the conventional wisdom that the economic crisis would inevitably undermine trust in others (e.g., van der Cruijsen et al., 2016).

In three studies, we investigated the effects of experiencing the economic crisis on generalized, interpersonal, and depersonalized in-group trust. The results of Studies 1 and 2 revealed that those most negatively affected by the crisis had lower levels of generalized trust and higher levels of interpersonal and depersonalized in-group trust. These effects, which occurred regardless of participants’ SES, also emerged when the influence of other socio-demographic and ideological factors (gender, age, marital status, political orientation, religiosity, and employment status) were controlled for. In addition, the results of an experimental study (Study 3) confirmed the causality of these relationships: the perception of personal economic threat linked to the crisis results in less trust in strange or unknown people (i.e., generalized trust) and greater trust in known or close individuals (i.e., interpersonal trust) and strangers who belong to a same group (i.e., depersonalized in-group trust).

These results contribute to the literature on the various socioeconomic determinants of trust by suggesting that economic crises do not affect the different forms of personal trust in the same way. Regarding generalized trust, it has been observed that economic inequality (e.g., Bjørnskov, 2007) or belonging to a particular social class (e.g., Alesina and La Ferrara, 2002) constitute some of its main determinants. Our findings expand this literature by showing that subjective perceptions of economic threat related to the crisis also exert a negative impact on this type of trust. Similarly, van der Cruijsen et al. (2016) found that individuals from a Dutch sample, who were customers of a bank that failed, showed lower levels of generalized trust than individuals who had not had such an experience. Thus, the decline in this type of trust among those who perceive a greater personal impact of the crisis, due to the greater psychological vulnerability and weakness that surrounds them, could represent a functional response aimed at avoiding the risks of being involved in an unfavorable transaction while socially interacting with a stranger. This decrease in generalized trust could undermine cohesion and adequate social functioning because generalized trust bridges nearby social relations and is fundamental in the creation of social capital (Putnam, 2000). In this sense, higher levels of generalized trust have been associated with more egalitarian societies, less crime, and less corruption (see Putnam, 1993).

Regarding interpersonal trust, the increase in this type of trust among those who experience the consequences of the crisis more intensely could be considered an adaptive response to a potentially threatening situation. Thus, people who were affected by the crisis could develop a greater protective tendency, restricting their trust to people who share a strong emotional commitment to them, most likely with the ultimate aim of protecting the resources threatened by the context of instability that characterizes the scenario of economic deprivation. Consistent with this idea, when faced with the economic crisis in Spain, individuals rely on family and friends as the main providers of social support to a greater extent than other groups or organizations (Centro de Investigaciones Sociológicas, 2010). Hence, close people constitute a fundamental shock absorber of the social and economic problems arising from the economic recession (Meil, 2011).

Concerning in-group trust, our finding of a greater tendency to trust people from the same social class among those most affected by the crisis complements previous research that finds an increase in this type of trust in the salience of social (i.e., thinking about the impact of a devastating hurricane) and economic crises (Kenworthy and Jones, 2009; Fritsche et al., 2017). Thus, our results reinforce the notion that this type of trust is a psychological group response aimed at restoring psychological equanimity in social situations that pose a threat to the *self*.

In the light of the foregoing, what psychosocial implications could arise from the decrease or increase in these manifestations of trust in times of crisis? Although trusting close or known people (i.e., interpersonal trust) or members of a same group (i.e., in-group trust) can provide greater stability and certainty to those who perceive themselves economically threatened in contexts of uncertainty, the decline in generalized trust may also constitute an obstacle for developing personal and social initiatives and for taking risks, thus hindering the possibility of accessing new growth opportunities (see Yamagishi et al., 1998). Thus, although higher levels of interpersonal trust could help minimize the risks stemming from a situation of personal vulnerability, this form of trust could be counterproductive in overcoming unfavorable socioeconomic conditions. This is so because particularized trusters, by circumscribing their trust to known and close people, tend either to be involved only in civic movements that involve individuals similar to themselves or tend not to participate civically at all (Uslaner and Conley, 2003), despite the fact that “participation in the larger society is important because it helps to build the bridges across groups that are essential to solving collective-action problems” (Uslaner and Conley, 2003, p. 334).

On the other hand, the increase in in-group trust could constitute a potentially constructive collective response to economic hardship (Fritsche et al., 2017). In this sense, Crepaz et al. (2017) indicated that in-group trust correlated positively with participation in conventional political actions (i.e., to vote). Moreover, previous studies found that lower trust in the neighborhood, which could be regarded as an example of in-group trust, is linked with lower self-perceived health (Bjornstrom, 2011). However, it is important to note that high intense group ties could hinder the development of generalized trust (Yamagishi, 2011). Therefore, the increase in this form of trust among those who are affected by economically threatening situations does not necessarily entail a positive response *per se*. We believe that future research is needed to obtain new evidence that sheds light on the consequences of interpersonal trust and in-group trust in contexts of economic vulnerability.

### Limitations and Future Directions

Although this study provides important new evidence relating to how the crisis affects the different types of trust, it is important to acknowledge several of its possible limitations as well as to suggest future research directions. First, the data were obtained from participants from a single country in crisis. It would be advisable for future researchers to determine whether our results hold in other countries strongly affected by periods of economic decline, either in a similar culture (e.g., Greece) or a different one (e.g., Ireland) while also investigating potential cultural differences. Second, the second step of the experimental manipulation used in Study 3 (i.e., the description of how someone close could be affected by what was described in the news) could entail a certain imbalance in terms of personal relevance in both conditions. For instance, the information described in the crisis-salience condition may be more personally relevant compared to the information described in the control condition. In any case, the results obtained showed that the mere fact of thinking of the Spanish context of economic crisis (vs. thinking of a non-economic issue) leads to a differential pattern of results in terms of trust. It would be interesting if future research developed new experimental designs to replicate these findings, while also controlling for personal relevance. Moreover, it would be advisable to implement longitudinal studies that allow for monitoring the potential changes in the levels of trust based on the changes of the socioeconomic conditions. Third, it would be interesting for future studies to include other measures of the personal impact of the crisis. It is true that in this research we have used a variety of measures, with consistent results. Nevertheless, it is clear that the personal affectation that results from experiencing crisis constitutes a complex psychological dimension. In addition to subjective measures of impact, other measures of objective nature (e.g., the cut of personal income, economic stagnation, etc.) might be included. Fourth, future studies could consider the possibility of assessing the different forms of trust by using other measures (e.g., the ones developed by Delhey et al., 2011), or considering other reference categories besides social class (e.g., race). In particular, of interest may be the study of the potential effect of the perceived impact of the economic crisis on the trust radius, i.e., the width of the circle of trusted individuals (Delhey et al., 2011; van Hoorn, 2014). Basically, the measurement of the radius of trust was conducted by calculating a coefficient for out-group trust (e.g., trust in people of another nationality, religion, etc.) and in-group trust (e.g., trust in people we know personally, of our neighborhood, etc.) and then subtracting the last coefficient (i.e., in-group trust) from the former (out-group trust) (Delhey et al., 2011; van Hoorn, 2014). Considering the parallelism between such measures and the ones included in this research (out-group trust would be similar to our measure of generalized trust and in-group trust to our measures of interpersonal and depersonalized in-group trust), one might wonder how the trust radius would change due to the impact of an economic crisis. Because our results suggest a lower inclination to trust in strangers and an increased tendency to trust in close people and those who belong to a shared group, it would be reasonable to expect a negative association between the perceived impact of the crisis and the trust radius. In other words, the radius of trust could be narrower in times of crisis and among those who are suffering its effects. Future studies should test this hypothesis. Lastly, a possible line of research could be represented by the study of the putative connection between the perceived impact of the crisis and the justification of the social system or status quo. Building on the system justification theory (Jost and Banaji, 1994), it has been suggested that the situations that represent a threat to the system may engender system-justifying processes (Kay and Friesen, 2011). Based on this, one might expect that citizens who perceive themselves to be adversely affected by the economic crisis – an example of systemic threat – are more inclined to justify the system. However, our data suggest that the reduction of generalized trust among those people may involve, indeed, that they would not justify the system because trust is an essential ingredient for the legitimacy of the institutions (Hough et al., 2010). Future research should clarify this issue.

## Conclusion

The results presented here entail the first empirical evidence that perceptions of personal impact of the economic crisis can differentially affect trust in others. In addition, these effects were found in both non-experimental and experimental studies. In conclusion, framed within a body of recent research that suggests that the perceptions of economic threat (e.g., perception of lower SES, fear of descending the social scale, perceived economic inequality) affect individuals’ attitudes and behavior (Fritsche and Jugert, 2017), our research contributes to filling a gap in the literature regarding the understanding of the relationship between macrosocial factors (i.e., economic crisis) and psychological processes (i.e., trust).

## Ethics Statement

The three reported studies, which are part of a research project approved by the Ethical Committee of the University of Granada, were carried out in accordance with the 1964 Declaration of Helsinki.

## Author Contributions

GN-C, IV-S, and MM conceived and designed the studies. GN-C conducted the studies. GN-C, IV-S, LL, and MM analyzed and interpreted the data, and drafted the manuscript.

## Conflict of Interest Statement

The authors declare that the research was conducted in the absence of any commercial or financial relationships that could be construed as a potential conflict of interest.

## Footnotes

1 Socioeconomic status did not moderate the effect of the perceived impact of the crisis on generalized trust, *b* = -0.038, *t* = -1.50, *p* = 0.135, 95% CI [-0.088, 0.012], interpersonal trust, *b* = 0.009, *t* = 0.26, *p* = 0.791, 95% CI [-0.056, 0.074], or on in-group trust, *b* = 0.007, *t* = 0.26, *p* = 0.797, 95% CI [-0.050, 0.065].

2 Socioeconomic status did not moderate the effect of the perceived impact of the crisis on any type of trust: generalized trust, *b* = -0.005, *t* = -0.38, *p* = 0.705, 95% CI [-0.033, 0.022]; interpersonal trust, *b* = 0.009, *t* = 0.54, *p* = 0.589, 95% CI [-0.023, 0.041]; and in-group trust, *b* = -0.001, *t* = -0.34, *p* = 0.973, 95% CI [-0.032, 0.031].

## References

[B1] Alesina A. La Ferrara E. (2002). Who trusts others? *J. Public Econ.* 85 207–234. 10.1162/003355399556269

[B2] Balliet D. Van Lange P. A. M. (2013). Trust, conflict, and cooperation: a meta-analysis. *Psychol. Bull.* 139 1090–1112. 10.1037/a0030939 23231532

[B3] Berg J. Dickhaut J. McCabe K. A. (1995). Trust, reciprocity, and social history. *Games Econ. Behav.* 10 122–142. 10.1006/game.1995.1027

[B4] Berggren N. Bjørnskov C. (2011). Is the importance of religion in daily life related to social trust? Cross-country and cross-state comparisons. *J. Econ. Behav. Organ.* 80 459–480. 10.1016/j.jebo.2011.05.002

[B5] Bjørnskov C. (2007). Determinants of generalized trust: a cross-country comparison. *Public Choice* 130 1–21. 10.1007/s11127-006-9069-1

[B6] Bjørnskov C. Méon P. G. (2013). Is trust the missing root of institutions, education, and development? *Public Choice* 157 641–669. 10.1007/s11127-013-0069-7

[B7] Bjornstrom E. (2011). The neighborhood context of relative position, trust, and self-rated health. *Soc. Sci. Med.* 73 42–49. 10.1016/j.socscimed.2011.05.014 21669482

[B8] Blanton H. Stuart A. E. VandenEijnden R. J. J. M. (2001). An introduction to deviance regulation theory: the effect of behavioral norms on message framing. *Pers. Soc. Psychol. Bull.* 27 848–858. 10.1177/0146167201277007

[B9] Buttrick N. R. Oishi S. (2017). The psychological consequences of income inequality. *Soc. Pers. Psychol. Compass* 11:e12304 10.1111/spc3.12304

[B10] Carballo-Cruz F. (2011). Causes and consequences of the Spanish economic crisis: why the recovery is taken so long? *Panoeconomicus* 3 309–328. 10.2298/PAN1103309C

[B11] Cardenas J. C. Carpenter J. (2008). Behavioural development economics: lessons from field labs in the developing world. *J. Dev. Stud.* 44 337–364. 10.1080/00220380701848327

[B12] Centro de Investigaciones Sociológicas (2010). Available at: http://www.cis.es/cis/export/sites/default/-Archivos/Marginales/2840_2859/2844/es2844.pdf

[B13] Crepaz M. M. L. Jazayeri K. B. Polk J. (2017). What’s trust got to do with it? The effects of in-group and out-group trust on conventional and unconventional political participation. *Soc. Sci. Q.* 98 261–281. 10.1111/ssqu.12271

[B14] Delhey J. Newton K. Welzel C. (2011). How general is trust in most people’? *Am. Sociol. Rev.* 76 786–807. 10.1177/0003122411420817

[B15] Evans A. M. Revelle W. (2008). Survey and behavioral measurements of interpersonal trust. *J. Res. Pers.* 42 1585–1593. 10.1016/j.jrp.2008.07.011

[B16] Fiske S. T. (2004). *Social Beings. A Core Motives Approach to Social Psychology.* New Jersey: Wiley.

[B17] Fiske S. T. Moya M. Russell A. M. Bearns C. (2012). “The secret handshake: Trust in cross-class encounters,” in *Facing Social Class: How Societal Rank Influences Interaction*, eds Fiske S. T. Markus H. R. (New York, NY: The Russell Sage Foundation Publications), 234–252.

[B18] Fitzpatrick J. Lafontaine M. F. (2017). Attachment, trust, and satisfaction in relationships: investigating actor, partner, and mediating effects. *Pers. Relationsh.* 24 640–662. 10.1111/pere.12203

[B19] Fritsche I. Jugert P. (2017). The consequences of economic threat for motivated social cognition and action. *Curr. Opin. Psychol.* 18 31–36. 10.1016/j.copsyc.2017.07.027 29221509

[B20] Fritsche I. Moya M. Bukowski M. Jugert P. de Lemus S. Decker O. (2017). The great recession and group-based control: converting personal helplessness into social class in-group trust and collective action. *J. Soc. Issues* 73 117–137. 10.1111/josi.12207

[B21] Fukuyama F. (1995). *Trust: The Social Virtues and the Creation of Prosperity.* New York, NY: Free Press.

[B22] Gheorghiu M. Vignoles V. Smith P. (2009). Beyond the United States and Japan: testing Yamagishi’s emancipation theory of trust across 31 nations. *Soc. Psychol. Q.* 72 365–383. 10.1177/019027250907200408

[B23] Gili M. Roca M. Basu S. McKee M. Stuckler D. (2013). The mental health risks of economic crisis in Spain: evidence from primary care centers, 2006 and 2010. *Eur. J. Public Health* 23 103–108. 10.1093/eurpub/cks035 23132877

[B24] Grusky D. B. Western B. Wimer C. (2011). *The Great Recession.* New York, NY: Russell Sage Foundation.

[B25] Helliwell J. F. Wang S. (2011). Trust and wellbeing. *Int. J. Wellbeing* 1 42–78. 10.5502/ijw.v1i1.9

[B26] Hernandez P. Minor D. (2015). *Political Identity and Trust. Harvard Business School Strategy Unit Working Paper No.* 16-012. Available at: http:nrs.harvard.edu/urn-3:HUL.InstRepos:17779602

[B27] Hogg M. A. (2007). “Social identity and the group context of trust: Managing risk and building trust through belonging,” in *Trust in Cooperative Risk Management: Uncertainty and Scepticism in the Public Mind*, eds Siegrist M. Earle T. C. Gutscher H. (London: Earthscan), 51–71.

[B28] Hough M. Jackson J. Bradford B. Myhill A. Quinton P. (2010). Procedural justice, trust, and institutional legitimacy. *Policing* 4 203–210. 10.1093/police/paq027 11658207

[B29] Igarashi T. Kashima Y. Kashima E. S. Farsides T. Kim U. Strack F. (2008). Culture, trust, and social networks. *Asian J. Soc. Psychol.* 11 88–101. 10.1111/j.1467-839X.2007.00246.x

[B30] Instituto Nacional de Estadística (2017a). Available at: http://www.ine.es/prensa/ecv-2017.pdf

[B31] Instituto Nacional de Estadística (2017b). Available at: http://www.ine.es/jaxiT3/Tabla.htm?t=4247

[B32] Instituto Nacional de Estadística (2017c). Available at: http://www.ine.es/jaxiT3/Tabla.htm?t=3996&L=0

[B33] Johnson N. Mislin A. (2011). Trust games: a meta-analysis. *J. Econ. Psychol.* 32 865–889. 10.1016/j.joep.2011.05.007

[B34] Jost J. T. Banaji M. R. (1994). The role of stereotyping in system-justification and the production of false consciousness. *Br. J. Soc. Psychol.* 33 1–27. 10.1111/j.2044-8309.1994.tb01008.x

[B35] Kaplan S. C. Levinson C. A. Rodebaugh T. L. Menatti A. Weeks J. W. (2015). Social anxiety and the big five personality traits: the interactive relationship of trust and openness. *Cogn. Behav. Therapy* 44 212–222. 10.1080/16506073.2015.1008032 25705989

[B36] Kay A. C. Friesen J. (2011). On social stability and social change: understanding when system justification does and does not occur. *Curr. Dir. Psychol. Sci.* 20 360–364. 10.1177/0963721411422059

[B37] Kenworthy J. B. Jones J. (2009). The roles of group importance and anxiety in predicting depersonalized ingroup trust. *Group Process. Intergroup Relat.* 12 227–239. 10.1177/1368430208101058

[B38] Knack S. Keefer P. (1997). Does social capital have an economic payoff? A cross-country investigation. *Q. J. Econ.* 112 1251–1288. 10.1162/003355300555475

[B39] Kramer R. M. (1999). Trust and distrust in organizations: emerging perspectives, enduring questions. *Annu. Rev. Psychol.* 50 569–598. 10.1146/annurev.psych.50.1.569 15012464

[B40] Kraus M. W. Piff P. Keltner D. (2009). Social class, sense of control, and social explanation. *J. Pers. Soc. Psychol.* 97 992–1004. 10.1037/a0016357 19968415

[B41] Lopez-Bernal J. A. Gasparrini A. Artundo C. M. McKee M. (2013). The effect of the late 2000s financial crisis on suicides in Spain: an interrupted time-series analysis. *Eur. J. Public Health* 23 732–736. 10.1093/eurpub/ckt083 23804080

[B42] López-Jiménez J. J. Renes V. (2011). Los efectos de la crisis en los hogares: nivel de integración y exclusión social. *Papeles Relaciones Ecosoc. Cambio Glob.* 113 189–199.

[B43] Lozano L. M. García-Cueto E. Muñiz J. (2008). Effect of the number of response categories on the reliability and validity of rating scales. *Methodology* 4 73–79. 10.1027/1614-2241.4.2.73 27403207

[B44] Lindström M. (2012). Marital status and generalized trust in other people: a population-based study. *Soc. Sci. J.* 49 20–23. 10.1016/j.soscij.2011.07.002

[B45] Marjanovic Z. Greenglass E. Fiksenbaum L. Bell C. (2013). Psychometric evaluation of the Financial Threat Scale (FTS) in the context of the great recession. *J. Econ. Psychol.* 36 1–10. 10.1016/j.joep.2013.02.005

[B46] Mauss I. B. Tamir M. Anderson C. L. Savino N. S. (2011). Can seeking happiness make people unhappy? *Emotion* 11 807–815. 10.1037/a0022010 21517168 PMC3160511

[B47] McAllister D. J. (1995). Affect and cognition-based trust as foundations for interpersonal cooperation in organizations. *Acad. Manag. J.* 38 24–59.

[B48] Meil G. (2011). *Individualización y Solidaridad Familiar.* Barcelona: Fundación La Caixa.

[B49] Moya M. Bearns C. Fiske S. T. (2011). “Social class, trust, and life aspirations,” in *Paper presented at 16th European Association of Social Psychology General Meeting*, (Stockholm.),

[B50] Moya M. Fiske S. T. (2017). The social psychology of the great recession and social class divides. *J. Soc. Issues* 73 8–22. 10.1111/josi.12201 26450754

[B51] Navarro-Carrillo G. Valor-Segura I. Moya M. (2018). Do you trust strangers, close acquaintances, and members of your in-group? *Soc. Indic. Res.* 135 585–597. 10.1007/s11205-016-1527-7

[B52] Oxfam Intermon (2016). *Una Economía al Servicio del 1%.* Available at: https://www.oxfamintermon.org/es/documentos/15/01/16/una-economia-al-servicio-del-1

[B53] Organization for Economic Cooperation and Development [OECD] (2014). *Rising Inequality: Youth and Poor Fall Further Behind - Income Inequality Update.* Paris: OCED.

[B54] Putnam R. (1993). *Making Democracy Work: Civic Traditions in Modern Italy.* Princeton: Princeton University Press.

[B55] Putnam R. (2000). *Bowling Alone: The Collapse and Revival of American Community.* New York, NY: Simon and Schuster.

[B56] Rousseau D. M. Sitkin S. B. Burt R. S. Camerer C. (1998). Not so different after all: a cross-discipline view of trust. *Acad. Manag. Rev.* 23 393–404. 10.5465/amr.1998.926617

[B57] Simpson J. A. (2007). Psychological foundations of trust. *Curr. Dir. Psychol. Sci.* 16 264–268. 10.1111/j.1467-8721.20007.00517.x

[B58] Schneider I. K. Konijn E. A. Righetti F. Rusbult C. E. (2011). A healthy dose of trust: the relationship between interpersonal trust and health. *Pers. Relationsh.* 18 668–676. 10.1111/j.1475-6811.2010.01338.x

[B59] Suárez-Álvarez J. Pedrosa I. Lozano L. M. García-Cueto E. Cuesta M. Muñiz J. (2018). Using reversed items in likert scales: a questionable practice. *Psicothema* 30 149–158. 10.7334/psicothema2018.33 29694314

[B60] Uslaner E. M. Conley R. S. (2003). Civic engagement and particularized trust: the ties that bind people to their ethnic communities. *Am. Polit. Res.* 31 331–360. 10.1177/1532673X03252528

[B61] van der Cruijsen C. de Haan J. Jansen D. J. (2016). Trust and financial crisis experiences. *Soc. Indic. Res.* 127 577–600. 10.1007/s11205-015-0984-8

[B62] van Hoorn A. (2014). Trust radius versus trust level: radius of trust as a distinct trust construct. *Am. Sociol. Rev.* 79 1256–1259. 10.1177/0003122414555398

[B63] Van Lange P. A. M. (2015). Generalized trust: lessons from genetics and culture. *Curr. Dir. Psychol. Sci.* 24 71–76. 10.1177/0963721414552473

[B64] Wilkinson R. Pickett K. (2009). *The Spirit Level: Why More Equal Societies Almost Always do Better.* London: Penguin.

[B65] Xin S. Xin Z. Lin C. (2016). Effects of trustors’ social identity complexity on interpersonal and intergroup trust. *Eur. J. Soc. Psychol.* 46 428–440. 10.1002/ejsp.2156

[B66] Yamagishi T. (2011). *Trust: The Evolutionary Game of Mind and Society.* New York, NY: Springer 10.1007/978-4-431-53936-0

[B67] Yamagishi T. Cook K. S. Watabe M. (1998). Uncertainty, trust and commitment formation in the United States and Japan. *Am. J. Soc.* 104 165–194. 10.1086/210005

[B68] Yamagishi T. Kikuchi M. Kosugi M. (1999). Trust, gullibility, and social intelligence. *Asian J. Soc. Psychol.* 2 145–161. 10.1111/1467-839X.00030

[B69] Yamagishi T. Yamagishi M. (1994). Trust and commitment in the United States and Japan. *Motiv. Emot.* 18 129–166. 10.1007/BF02249397

